# Change of collective orientation through an interprofessional training with medical students and student nurses depending on presence and professional group

**DOI:** 10.1186/s12909-021-02804-7

**Published:** 2021-07-03

**Authors:** M Flentje, V Hagemann, G Breuer, P Bintaro, H Eismann

**Affiliations:** 1 Department of Anaesthesiology and Intensive Care Medicine, Hannover Medical School, Carl- Neuberg-Strasse 1, 30625 Hannover, Germany; 2 Faculty of Business Studies and Economics, University of Bremen, Enrique-Schmidt-Strasse 1, 28359 Bremen, Germany; 3 Department of Anaesthesiology, REGIOMED Kliniken, Ketschendorfer Strasse 33, 96450 Coburg, Germany; 4 Department of Nephrology, Hannover Medical School, Carl-Neuberg-Strasse 1, 30625 Hannover, Germany

**Keywords:** Patient fall, Non-technical skill, Interprofessional teamwork, Collective Orientation, Student learning, Simulation

## Abstract

**Background:**

Teamwork is an important success factors for patient treatment. The willingness of a healthcare provider to work in a team can be descripted with the construct of “Collective Orientation” (CO). The level of CO can be trained and is related to team performance. In this study, we investigated the effect of a simulator-based interprofessional training on the subject of patient fall in a hospital setting upon participations CO. To evaluate whether the course could be integrated into a longitudinal education concept, the participants were medical students and student nurses. Since effects of simulations can be influenced by the perceived reality, the results were measured as a function of Presence.

**Method:**

In this observation study, 62 medical students and student nurses took part in six one-day interprofessional simulation trainings with the topic patient fall. The primary outcome was the mean difference between the CO measured immediately before (T1) and after the training (T2). The Presence of the participants was measured by questionnaire immediately after the course (T2).

**Results:**

Cronbach´s alpha for all scales and measurement points was higher than 0.69. CO increases over all professional groups from *M* = 3.42 (*SD* = 0.39) to *M* = 3.68 (*SD* = 0.54) significantly (*p* < .00; *r* = .5). Only the subscale “Dominance” in the professional group of the student nurses did not increase significantly. There was no correlation between Presence and the change in CO.

**Conclusion:**

The questionnaires of CO and Presence can be applied to medical students and student nurses. The simulation course with the topic patient fall influences the CO and can be integrated in a longitudinal curriculum of teamwork training. The subscale “Dominance” of student nurses did not change. Preparatory learning units may increase the effects. The perceived reality of the scenario is not a main success factor.

## Background

Successful patient treatment does not only require extensive medical knowledge and implementation of technical procedures but also other skills. Teamwork is one of these success factors and therefore cannot be called a “nice to have” quality. Since the 1990 s, studies have been pointing the positive effect of teamwork [1] and recent metanalyses recommend the perception of teamwork for the benefit of patients [2]. In the context of incident management, teamwork is described as one of the non-technical skills required for coping [3]. Teams can be defined as identifiable social work units consisting of two or more people. These characteristics include social interaction, shared and valued goals, a discrete lifespan, distributed expertise and assigned roles and responsibilities [4, 5].

In line with the importance of teamwork described above, successful teamwork is requested as a competence in the education curriculum for medical students and student nurses. In Germany, the NKLM (“Nationaler Kompetenzbasierter Lernzielkatalog Medizin”) was developed to describe the expected competencies of medical students [6]. Chapter 8 describes the competencies and learning objectives for the role as a team member (e.g. “They participate actively and constructively in the teamwork for common tasks.”). For the nurse education, an examination regulation of the federal government applies [7]. For example, Section III.3 of the practical training describes the competence “collaborating in interdisciplinary teams with other occupational groups”. It is not specified with which training method the competence goal has to be achieved.

Curriculum development in medical education should be well structured to achieve high efficiency. One of the well-known conceptual frameworks is the six-step Kern-cycle for medical curriculum development [8]. We took steps one to three (“problem identification”, “targeted needs assessment” and “goals and objectives”) as complied by the background described above. In this study we focused on step four (“educational strategies”) and its evaluation.

Diskrell et al. used the term Collective Orientation (CO) to describe the willingness of an individual to work in a team [9]. CO consists of the subscales “Affiliation” and “Dominance”, can be measured by questionnaire [10] and can be changed by training [4]. A high “Affiliation describes the ability to work both in a goal-oriented manner and with a higher regard for others in a team. A high degree of “Dominance” demonstrates a priority in having power and control. A high value of CO is associated with higher team performance [9, 11, 12].

Simulation-based training is a recognized method for training non-technical skills [13]. We developed an interprofessional simulation course and already evaluated this concept on the level of subjective gain of cooperation [14]. This course is the basis of this study.

With regard to the teaching method “simulation”, the perceived degree of reality of the participants is discussed as a factor influencing the learning effect. The trainee should consider the training as being adequate for its purpose and the consequences of his actions taken are represented as they would occur in a real-life situation [15]. Several studies found a positive correlation between perceived degree of reality and learning outcomes [16]. Various theoretical models are available to describe this perceived degree of reality, as for example a fiction contract [17] and immersion [18]. In our study we used the model “Presence Scale for Labbased Microworld Research” (PLBMR), which is available validated in German language [19] and adapted to the health care context [20].

The aim of this study was to assess the impact of an interdisciplinary simulation training of medical students and student nurses on the willingness to work in a team. The results are used to assess a necessary integration of this training into a curriculum.

The hypotheses were: the scales of Collective Orientation and PLBMR are applicable to German 6th year medical students (a) and 2nd /3rd year student nurses (b). CO of medical students (c) and student nurses (d) is influenced by means of simulation training. The training effect and Presence is dependent on the profession (e) and the change of CO depends on the perceived Presence in the scenario (f).

## Methods

### Study design

The study was designed as a pre/post study using survey methodology with participants from sixth-year medical students and student nurses (second or third year). The first measurement (T1) was taken immediately prior to the training, and the second (T2) was completed immediately after the course. The items for assessing the Presence were also completed after the course.

Since we have not found any gender- and age-specific differences in other studies (similar number of cases) on the CO [21], we did not generate a hypothesis regarding this topics. Concluding from the hypotheses we asked for the professional group as demographic data. The ethics committee of Hannover Medical School approved the study (no. 8677_BO_K_2019).

### Setting and Population

The professional groups were equally represented in each course with ten participants per training day. Two courses were conducted with one additional student nurse or one additional medical student, respectively. The additional participants were each assigned to one scenario.

The medical students were required to have completed the second part of the German medical examination. In this phase of education, practical training in everyday clinical practice takes place. The student nurses were at the end of their second or in the third year of education. In the education of nursing the participants are regularly deployed in clinical practice beginning at their first year. These requirements ensured that there is sufficient practical experience and an awareness of existing interprofessional conflicts in daily routine.

The sixth-year medical students were invited by mail via a distribution list of the local university. They were informed about the training content (interprofessional training by means of simulation). Beforehand, it was discussed with the dean of student affairs that participants will not be credited with a day of absence for their training place. The students could register on a scheduled course via mail. The student nurses were informed personally during lessons and could then register voluntarily on a participation list.

### Training course

The training course “patient fall” was designed to train the non-technical skills communication and teamwork in a relevant clinical situation. The topic of the scenarios “patient fall” was chosen very generally, so that both cause and treatment are not clear at the beginning (in contrast to e.g. resuscitation). This lack of clarity should make intensified communication necessary to cope with the situation. The scenarios took place on a simulated ward with basic emergency equipment readily available.

Since non-technical skills are not taught in either nursing school or medical education, a lecture on “crisis resource management” was given during the first part of the course. Further theoretical learning units were “conflicts between professional groups” and “patient fall - causes and treatment”. After familiarization with the patient simulator and the simulation environment, the scenarios were carried out. Table 1 shows the agenda of the course.

**Table 1:** Progress of the course “Patient Fall”. Each scenario was managed by on student nurse and on medical student in the active role [14]

Time	Topic	Method of teaching
15 min	welcome / introduction	Discussion
30 min	introduction to crew resource management (CRM)	Lecture
45 min	experience with conflicts between professional groups	Discussion
30 min	pathophysiology of a patient fall	Lecture
30 min	familiarisation with the simulator	Presentation
30 min	patient fall: Femoral fracture, head wound	Simulated scenario
30 min	patient fall: Dementia	Simulated scenario
30 min	patient fall: Hypoglycemia	Simulated scenario
30 min	patient fall: Getting up to soon after spinal block	Simulated scenario
30 min	patient fall: Anaphylaxis	Simulated scenario
30 min	discussion and course completion	Discussion

Each scenario was conducted by one student nurse and one medical student - each in an active role. At the end of the scenario, the team should have an idea of a further treatment plan for the patient (e.g. CT-scan). In contrast to the initially published course [14], the debriefings were conducted according to the TeamGAINS concept [22] and a video-debriefing was used.

### Collective Orientation

The Collective Orientation was assessed at two measuring points using paper-based questionnaires (T1: between study information and start of the course, T2: after completion of the course). The questionnaire consists of two scales with ten items for “affiliation” and six items for “dominance” [22]. The complete questionnaire is shown in Table 2. Items marked with (R) have to be reverse scored. The mean of all values results in the CO.

**Table 2:** Items of Collective Orientation. In the German version two items were exchanged for the dominance. For the affiliation two were added and one was removed. Items marked with a (R) are negatively worded and have to be reversed-scored

Collective Orientation: Subscales and Items	
Introduction: In the following you will see a series of statements. The concern is with your own option. Therefore, there are no “right” or “wrong” answers. Answer the question such that they best apply to you. Please respond to the statements in terms of your personal attitude. Items could be answered on a 5-point-Likert scale from 1 (“stimme gar nicht zu” / “I totally disagree”) to 5 (“stimme voll zu” / “I totally agree”).”	
**German Version**	**English Version**
Zugehörigkeit	Affiliation
Ich finde die Arbeit an Teamprojekten sehr zufriedenstellend.	I find working on team projects to be very satisfying.
Ich würde eher selbst handeln als auf den Input von anderen zu warten (R).	I would rather take action on my own than to wait around for others`
Ich bevorzuge es eine Aufgabe von Anfang bis Ende durchzuführen, ohne die Unterstützung von anderen (R).	I prefer to complete a task from beginning to end with no assistance from others.
Teams arbeiten normalerweise sehr effektiv.	Teams usually work very effectively.
Ich denke es ist normalerweise besser den Stier bei den Hörnern zu packen und etwas selber zu machen, als darauf zu warten Input von anderen zu bekommen(R).	I think it is usually better to take the bull by the horns and do something yourself, rather than wait to get input from others.
Bei den meisten Aufgaben würde ich eher allein arbeiten, als Teil einer Gruppe zu sein (R).	For most tasks, I would rather work alone than as a part of a group.
Ich kann normalerweise mehr leisten, wenn ich für mich alleine arbeite (R).	I can usually perform better when I work on my own.
Ich finde, dass es meist produktiver ist für mich alleine zu arbeiten als mit anderen (R).	I find that it is often more productive to work on my own
Ich arbeite gerne mit anderen zusammen. (only German version)	I find it easy to negotiate with others who hold a different viewpoint than I hold. (only English version)
Ich finde es nicht gut sich auf andere Teammitglieder verlassen zu müssen (only German version) (R)	I always ask for information from others before making any important decision. (only English version)
Dominanz	Dominance
Wenn ich anderen Teammitgliedern nicht zustimme, neige ich dazu meinem eigenen Bauchgefühl zu folgen (R).	When I disagree with other team members, I tend to go with my own gut feelings.
Wenn ich eine andere Meinung als ein anderes Teammitglied habe, versuche ich normalerweise bei meiner eigenen Meinung zu bleiben (R).	When I have a different opinion than another group member, I usually try to stick with my own opinion.
Es ist wichtig bei der eigenen Meinung zu bleiben, gerade wenn andere um dich herum versuchen dich zu einer Änderung zu bewegen (R).	It is important to stick on your own decision, even when others around are trying to get you to change.
Wenn andere widersprechen, ist es wichtig standzuhalten und nicht nachzugeben (R). (only German version)	
Ich finde auch bei Teamarbeiten sollte man immer das tun, was man selbst für richtig hält. (R) (only German version)	When solving a problem, it is very important to make your own decision and stick by it. (only English version)
Wenn ich von etwas überzeugt bin bleibe ich bei meiner Meinung, egal was andere Teammitglieder dazu sagen (R).	When others disagree, it is important to hold one´s own ground and not give in.

### Presence

The scale “Presence Scale for Lab-based Microworld Research” (PLBMR) was developed by Frank et al. to describe the Presence during simulation scenarios [19]. The items are rated with a 6-Point-Likert-scale from 1 (“totally disagree”) to 6 (“totally agree”). Table 3 shows all six items. Items marked with (R) are negatively worded and have to be reversed scored. The mean of all values results in the measured Presence.

**Table 3:** Items of Presence Scale for Lab-based Microworld Research (PLBMR) for German and English trainees [19]. Items with an (R) are negatively worded and have to be reversed-scored

German	Translated into English
Introduction: You will now read a series of statements that each describe their perception of the simulation world. Indicate to what extend the statement applies. The size of the number correlates with the approval. Three examples were given with big approval and big dislike. Items could be answered on a 6-point-Likert-scale from 1 (“trifft nicht zu”/ “I totally disagree”) to 6 (“trifft vollständig zu”/ “I totally agree”).	
Ich habe mich als Teil der Simulationswelt gefühlt.	I felt like I was part of the Simworld.
Die Simulationswelt hat bei mir Emotionen (z.B. Ärger, Traurigkeit, Zufriedenheit) ausgelöst.	The simulation world triggered my emotions (e.g. anger, sadness, satisfaction)
Die Arbeit mit der Simulationswelt war für mich zufriedenstellend.	Working in the simulation world was satisfying for me.
Während ich in der Simulation war, habe ich zwischenzeitlich vergessen, dass ich an einer Studie teilnehme.	While operating the simulation, I forgot for the time being that I was taking part in a study.
Die Arbeit in der Simulationswelt war für mich langweilig (R).	Working in the simulation world was boring for me.
Während ich in der Simulation war, bin ich gedanklich in die Simulationswelt abgetaucht.	While operating the simulation, my thoughts became immersed in the simulation world.

### Statistical analysis

Demographic data were analyzed in a descriptive manner. For testing hypothesis (a) and (b), the reliability of the scales was determined by Cronbach´s alpha. In order to test hypothesis (c) and (d) a Wilcoxon-test for dependent samples and a Mann-Whitney-Test for independent samples was conducted. For differences between the occupational groups in presence, a t-test for unrelated samples was applied (e). In order to test hypothesis (f) - whether a change in CO is dependent on the perceived presence - a Pearson correlation was calculated. We assumed a *p* < .05 as being statistically significant. As an effect size, r was calculated. All data was initially recorded in Excel (Microsoft, Redmond, USA) and calculated using SPSS (Version 26, IBM Corporation, USA).

## Results

A total of 62 participants completed the questionnaires. Each professional group was represented by 31 participants.

### Reliability of the scales

In order to test hypothesis (a) and (b) Cronbach´s alpha was calculated. The result for the CO questionnaire was 0.74 (T1) and 0.79 (T2). No item reduction let to a substantial increase in Cronbach´s alpha. Subdivided by subscales and measuring time, Cronbach´s alpha was 0.78 (T1) and 0.80 (T2) for the subscale “Affiliation” and 0.69 (T1) and 0.75 (T2) for the subscale “Dominance”. For the questionnaire PLMBR, Cronbach´s alpha was 0.70. An item reduction did not improve the values.

### Pre-post difference in Collective Orientation

In order to test hypothesis (c) and (d), the change of CO was evaluated by calculation the difference in CO before (T1) and after (T2) training with regard to the professional groups. Overall, CO changed form *M* = 3.42 (*SD* = 0.39) to *M* = 3.68 (*SD* = 0.54) significantly (*p* < .00; *r* = .5). Over all participants, the subscale “Affiliation” was *M* = 3.37 (*SD* = 0.48) before and *M* = 3.63 (*SD* = 0.54) after training. The difference was significant (*p* > .00; *r* = .53). For the subscale “Dominance” the value changed from *M* = 3.51 (*SD* = 0.59) to *M* = 3.68 (*SD* = 0.59). The difference was also significant (*p* < .000; *r* = .26).

For medical students the values changed significantly (*p* < .00; *r* = .31) for affiliation from *M* = 3.53 (*SD* = 0.42) to *M* = 3.81 (*SD* = 0.47) and for dominance from *M* = 3.68 (*SD* = 0.49) to *M* = 3.88 (*SD* = 0.52) significantly (*p* < .01; *r* = .31).

**Fig. 1:**
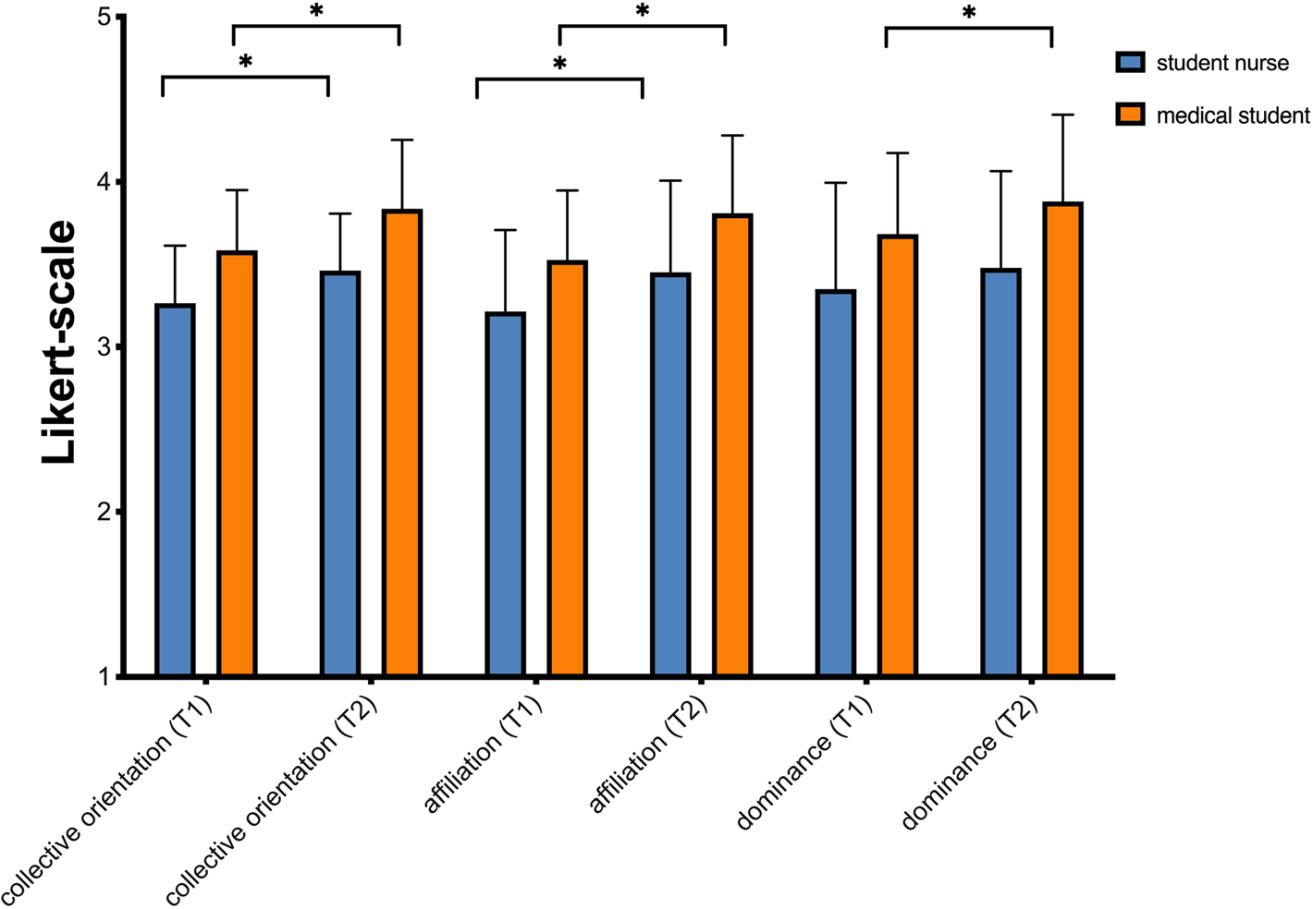
Differences in Collective Orientation and subscales prior (T1) and after training (T2) for student nurses and medical students. *indicates the significant difference. The questions were rated on a 5-point-Likert-type scale from 1 (“trifft nicht zu” / “I totally disagree”) to 5 (“trifft vollständig zu” / “I totally agree”)

For student nurses “Affiliation was *M* = 3.21 (*SD* = .5) at T1 and *M* = 3.45 (*SD* = .56) at T2 with a significant change (*p* = .00; *r* = .25). The change of the subscale dominance from *M* = 3.45 (*SD* = .65) to *M* = 3.48 (*SD* = .59) was not significant (*p* = .110). All results are shown in Fig. 1.

### Presence

**Fig. 2:**
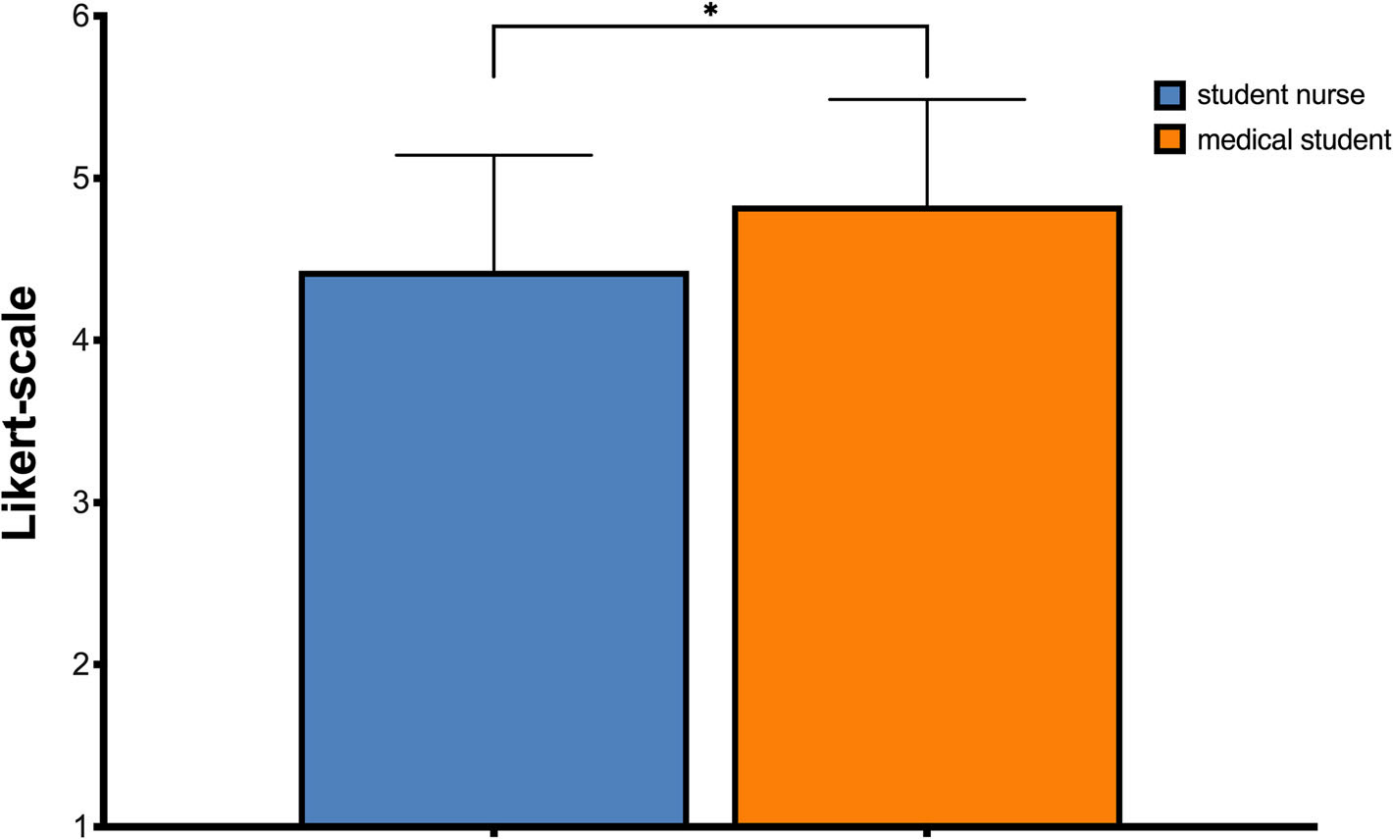
Rating of the Presence by the participants after the training. The difference is significant with a small effect size (0.2)

Over all participants, the presence was *M* = 4.63 (*SD* = 0.71). As shown in Fig. 2, medical students evaluated the Presence with *M* = 4.83 (*SD* = 0.65), student nurses with *M* = 4.43 (*SD* = 0.71). The Presence rated by the medical students was significantly higher (*p* < .029; *r* = .2).

**Fig. 3:**
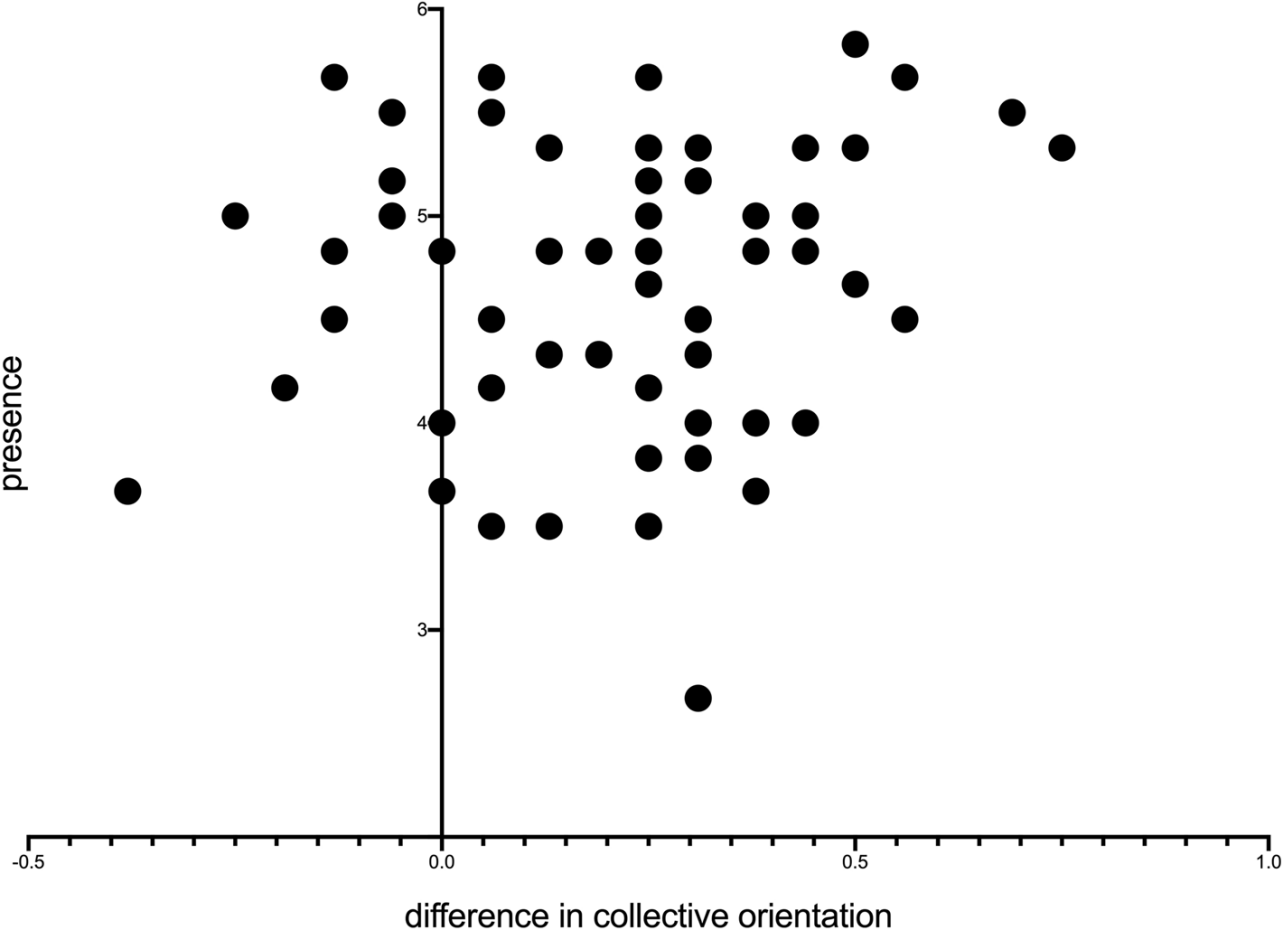
Change in Collective Orientation (CO) in relation to Presence (Pearson correlation 0.152). There is no systematic association between the change in the CO and the Presence of the participants (*n* = 62)

To test hypothesis (d), whether the effect of CO is correlated to the perceived Presence the correlation coefficient was calculated according to Pearson. Figure 3 shows that there is no systematic association between the changes in CO and the perceived Presence (Pearson correlation = 0.152, *p* > .05).

## Discussion

The aim of this study was to investigate the influence of interprofessional simulation training on the willingness to work in a health care team. The used measurement instruments were validated for their applicability in the target group. With values for internal consistencies above 0.7 for the subscales as well as for the complete questionnaires the required quality markers were fulfilled. Both questionnaires are applicable in the target group.

As a result of the training, CO increases in both professional groups. For further interpretation the subscales will be taken into account. In both professional groups “Affiliation” increases significantly with a medium effect. The medium effect of the change is satisfying with the investment of a one-day course. Changes in the work culture - of which teamwork is certainly one - are influenced by previous experiences and are rather difficult [23]. The participants were able to handle critical situations in the scenarios and recognized - both in an active role and as observer of other teams - how teamwork is a crucial success factor. Own experiences and the relevance of the topic are known determining factors for learning success [24]. The effect on the CO does not seem to depend on professional experience, as we have made the same observation with experienced anesthesiologists before [21]. As observed in a previous study, the effect of increased “Affiliation” does not occur, when the nurse is simulated by a physician [25]. This simulation artifact is apparently not correctable and influences the attitude of belonging to the team.

The values of “Dominance” of the medicine students changed to a cooperative working style with a medium effect. Low values of “Dominance” represent the tendency to power and control. We interpret the increase as a gain in students’ confidence in the professional skills of the nurses. In this course the students were in one learning group with nurses the first time outside clinical everyday life. Mental models could be synchronized and the abilities of nurses became more transparent. Dominance did not change among the student nurses. This may be favored by the topic of the course. In an emergency situation, the physician is responsible for the treatment process and will strive to prescribe the treatment plan. Nurses can influence the process of treatment by using incident management methods like e.g., “speaking up”. It could not be ensured that this method was taught and trained.

The results of the CO show that the course can be integrated into a longitudinal teaching concept on teamwork. Further effects can possibly be caused or strengthened by pre-course preparation with e.g. theoretical content. An example shows the IPE (interprofessional education) program of the McGill University [26]. The sequences of the steps “role clarification”, “team function” and the “patient care and simulation” described there, are also integrated in our course, although much shorter.

CO describes the willingness to work in a team. This definition differs from other assessments that describe good teamwork. The framework of “Observing and Anaesthetists’ Non-Technical Skills” rates “teamwork” as one competence of four non-technical skills [3]. The “Assessment of Interprofessional Team Collaboration Scale” (AITCS) assesses collaboration in health care systems in practice settings. Both systems have their own target group, so the AITCS system involved patients and their relatives. Both systems require a before-after consideration to describe the change in teamwork. In our setting, a recurred contact with the participants was not possible. Hence, the method of measuring CO offers a good alternative for testing the effectiveness of a didactic concept.

Our results show a difference between the professional groups in the values of Presence, but this had only a small effect. MacLean et al. also found that the Presence varies between individuals and changes differently in a series of simulation [16]. Student nurses have more practical experience with real patients during their training. The medical students have a higher proportion of theoretical teaching and have practical trainings without direct patient contact (e.g., histology classes). Due to the daily contact with patients, it might have been more difficult for the student nurses to get involved into the simulation. Since the measured values of both professional groups were high, we interpret the difference as not relevant for the course.

There was no correlation between Presence and increase of CO. This result is similar to a study in which we observed experienced anaesthesia nurses and physicians and the change in CO as a function of Presence [21]. In both studies, the professional groups were represented by members of their professional field. The role in the scenario corresponded to the intended role in the professional practice and was therefore not part of the simulation world. This might be a reason, why Presence is not important with regard to the competence goal of teamwork. However, all given values were also high, so that there could be a critical lowest level of Presence for CO-change. Furthermore, studies show different relevance of the perceived reality of the participants to the learning effect. There are studies that show no influence [27] as well as works that describe it positively [28]. Further studies about the learning target should be conducted. The question of a lower value can be particularly interesting for further study for the question how much resources must be invested in the scenario reality.

### Limitations

There are some limitations associated with this study. Due to the framework conditions, such as video recording for debriefing, the study was limited to voluntary participants. This may result in a biased pre-selection of the participants. Participants, who are critically opposed to an interprofessional cooperation or had already conflict-loaded experiences, could be deterred by the topic. It therefore remains open whether the persons who have lower CO-values in a cohort were also represented in the course. It would be interesting, how the course could influence this potential subgroup.

 Our study was a monocentric study with participants of a university hospital in Germany. There might be a culture and training environment, which influences the results. In comparisons with other studies, this and the details of the course (e.g., type of debriefing) must be taken in account. Further studies should investigate whether the effect of increased CO is implemented in everyday clinical practice and whether the effect is maintaining over time.

In every study, the recommendation for a control group seems comprehensible. It seems difficult to create a control group for the study group in this setting. If the same course is organized separately (monoprofessional control group), who then carries out the procedures of the other professional group? Providers have to play the other professional group or the scenarios have to be adapted. Both brings aspects to the control group that make it unusable.

## Conclusions

The CO and Presence scales meet the quality criteria for use by medical students and student nurses. CO increases by an interprofessional simulation course with the topic “patient fall” and the general conditions video- and TeamGAINS-debriefing. The course can be integrated in a longitudinal curriculum to train teamwork. The subscale “dominance” of the student nurses was the only one that was not influenced. The interprofessional simulation course “patient fall” is for the learning target teamwork. Preparatory learning units, as already shown in interprofessional curricular on interprofessional teamwork, may also support an effect here. Presence differs in the professional groups only with small effect. The effects of CO are independent of the measured Presence, which according to our interpretation is also supported by the realistic occupation of the professional groups in the scenario by the participants.

## Data Availability

Upon request, the author will provide the original dataset of the study.

## Abbreviations

CO: Collective Orientation
PLBMR: Presence Scale for Lab-based Microworld Research.
TeamGAINS: Guided team self-correction, advocacy-inquiry and systemic-constructivist techniques

## References

[1] Fagin CM (1992). Collaboration between nurses and physicians: no longer a choice. Acad Med.

[2] Schmutz JB, Meier LL, Manser T (2019). How effective is teamwork really? The relationship between teamwork and performance in healthcare teams: a systematic review and meta-analysis. BMJ Open.

[3] Flin R, Patey R, Glavin R, Maran N (2010). Anaesthetists’ non-technical skills. Br J Anaesth.

[4] Salas E, Rosen MA (2013). Building high reliability teams: progress and some reflections on teamwork training. BMJ Qual Saf.

[5] Salas E, Rosen MA, King H (2007). Managing teams managing crises: principles of teamwork to improve patient safety in the Emergency Room and beyond. Theoretical Issues in Ergonomics Science.

[6] Nationaler Kompetenzbasierter Lernzielkatalog Medizin. In. Fakultätentag D, editor. 2015.

[7] Ausbildungs-. und Prüfungsverordnung für die Pflegeberufe (Pflegeberufe-Ausbildungs- und Prüfungsverordnung - PflAPrV). In: Verbraucherschutz BfJu, editor. BGBl. I S. 1018 ed2018.

[8] Thomas PA, Kern DE, Hughes MT, Chen BY. Curriculum Development for Medical Education. 3 ed. Baltimore2016.

[9] Driskell JE, Salas E (1992). Cellective Behavior and Team Performance. Hum Factors.

[10] Driskell JE, Salas E, Hughes S (2010). Collective Orientation and Team Performance: Development of an Individual Differences Measure. Hum Factors.

[11] Hagemann V, Kluge A (2017). Complex Problem Solving in Teams: The Impact of Collective Orientation on Team Process Demands. Front Psychol.

[12] Hagemann V, Ontrup G, Kluge A. Collective orientation and its implications for coordination and team performance in interdependent work contexts. Team Performance Management: An International Journal. 2020;ahead-of-print(ahead-of-print).

[13] Gaba DM, Howard SK, Fish KJ, Smith BE, Sowb YA (2001). Simulation-Based Training in Anesthesia Crisis Resource Management (ACRM): A Decade of Experience. Simulation Gaming.

[14] Flentje M, Müßel T, Henzel B, Jantzen J-P. Simulation a patient´s fall as a means to improve routine communication: Joint training for nursing and fifth-year medical students. GMS Journal for Medical Education. 2016;33(2). 10.3205/zma001018 PMC4895856 27280130

[15] Hagiwara MA, Backlund P, Soderholm HM, Lundberg L, Lebram M, Engstrom H (2016). Measuring participants’ immersion in healthcare simulation: the development of an instrument. Adv Simul (Lond).

[16] MacLean S, Geddes F, Kelly M, Della P (2019). Realism and Presence in Simulation: Nursing Student Perceptions and Learning Outcomes. J Nurs Educ.

[17] Dieckmann P, Gaba D, Rall M (2007). Deepening the Theoretical Foundations of Patient Simulation as Social Practice. Simulation in Healthcare.

[18] Dede C (2009). Immersive Interfaces for Engagement and Learning. Science.

[19] Frank B, Kluge A. Development and first validaton of the Presence Scale (PLBMR) for lab-based microrworld research. 15th International Conference on Presence; Vienna, Austria: Facultas WUV Universitätsverlag; 2014. p. 31–42.

[20] Hagemann V, Herbstreit F, Kehren C, Chittamadathil J, Wolfertz S, Dirkmann D (2017). Does teaching non-technical skills to medical students improve those skills and simulated patient outcome?. Int J Med Educ.

[21] Flentje M, Eismann H, Sieg L, Hagemann V, Friedrich L (2020). Impact of Simulator-Based Crisis Resource Management Training on Collective Orientation in Anaesthesia: Pre-Post Survey Study With Interprofessional Anaesthesia Teams. J Med Educ Curric Dev.

[22] Kolbe M, Weiss M, Grote G, Knauth A, Dambach M, Spahn DR (2013). TeamGAINS: a tool for structured debriefings for simulation-based team trainings. BMJ Qual Saf.

[23] Smith Martin E (2003). Changing an organisation’s culture: correlates of success and failure. Leadership Organization Development Journal.

[24] Kolb DA. Experiential Learning: Experience as the Source of Learning and Development. Pearson Education; 2015.

[25] Eismann H, Palmaers T, Tsvetanov S, Hagemann V, Flentje M. Changes of collective orientation through a medical student’s anaesthesia simulation course – simulation-based training study with non-technical skills debriefing versus medical debriefing. BMC Medical Education. 2019;19(1). 10.1186/s12909-019-1765-x PMC6727403 31488119

[26] Interprofessional Education Curricullum. Mc Gill University; Available from: https://www.mcgill.ca/ipeoffice/ipe-curriculum/ipe-courses-objectives.

[27] Mills BW, Carter OB, Rudd CJ, Claxton LA, Ross NP, Strobel NA (2016). Effects of Low- Versus High-Fidelity Simulations on the Cognitive Burden and Performance of Entry-Level Paramedicine Students: A Mixed-Methods Comparison Trial Using Eye-Tracking, Continuous Heart Rate, Difficulty Rating Scales, Video Observation and Interviews. Simul Healthc.

[28] Stevens JA, Kincaid JP (2015). The Relationship between Presence and Performance in Virtual Simulation Training. Open Journal of Modelling Simulation.

